# Amino Acid Intakes and Dietary Sources in a Nationally Representative Sample of Older Adults in Ireland: Findings from the National Adult Nutrition Survey (NANS)

**DOI:** 10.3390/nu18030487

**Published:** 2026-02-02

**Authors:** Aoife Burke, Emma O’ Sullivan, Linda Giblin, Anne P. Nugent, Albert Flynn, Breige A. McNulty, Laura Kehoe, Michael Callanan, Janette Walton

**Affiliations:** 1 Department of Biological Sciences, Munster Technological University, T12 P928 Cork, Ireland; aoife.burke2@mymtu.ie (A.B.); emma.osullivan@mtu.ie (E.O.S.); laura.kehoe@mtu.ie (L.K.); michael.callanan@mtu.ie (M.C.); 2 Teagasc Food Research Centre, Moorepark, Fermoy, P61 C996 Cork, Ireland; linda.giblin@teagasc.ie; 3 Institute for Global Food Security, Queen’s University Belfast, Belfast BT9 5DL, UK; a.nugent@qub.ac.uk; 4 Institute of Food and Health, School of Agriculture and Food Science, University College Dublin, D04 V1W8 Dublin, Ireland; breige.mcnulty@ucd.ie; 5 School of Food and Nutritional Sciences, University College Cork, T12 K8AF Cork, Ireland; a.flynn@ucc.ie

**Keywords:** amino acids, food composition, protein, sarcopenia, frailty, ageing, dietary surveys

## Abstract

**Background/Objectives**: The global population is ageing rapidly, with projections indicating that there will be over two billion individuals aged ≥60 years by 2050. Sarcopenia and frailty are major age-related syndromes associated with loss of muscle mass, reduced strength, and increased vulnerability, for which adequate protein and amino acid intake are key preventive factors. However, nationally representative data on dietary amino acid intakes and sources among older adults are lacking, particularly in Europe. **Methods**: This study aimed to address this gap by updating the Irish Food Composition Database (IFCD) (2011) with amino acid composition data and estimating amino acid intakes and dietary sources in older adults in Ireland (≥65 years) using data from the National Adult Nutrition Survey (2008–2010; n = 226). **Results**: Mean total amino acid intake was 76.2 g/day (1.0 g/kg body weight/day). Intakes of all essential amino acids were above the US Institute of Medicine (IOM) recommendations, with no significant differences observed between sexes or age groups (65–74 y, 75+ y). ‘Meat and meat dishes’ were the principal contributors to amino acid intake (28–47%), followed by ‘breads and rolls’, ‘milk and yoghurt’, and ‘fish and fish dishes’. **Conclusions**: This study provides the first nationally representative estimates of amino acid intakes in older adults in Europe, establishing a baseline for future dietary surveillance and informing protein quality assessment amid dietary transitions toward plant-based foods.

## 1. Introduction

Population ageing is increasing at an unprecedented rate and it is predicted that by 2050 that there will be over two billion people globally over the age of 60 years, with 426 million over the age of 80 years [1,2].

In addition to the prevalence of most major non-communicable diseases of adulthood increasing with advancing age, other factors determining health in later life have also been identified, with two later-life syndromes, sarcopenia and frailty, being synonymous with age-related health issues [3,4]. Sarcopenia, a progressive muscle disease, is characterised by a decline in muscle mass and strength, whereas frailty is a distinct condition characterised by diminished strength and endurance and an increased vulnerability to stress caused by a decline in many physiological functions during ageing [3,5,6,7]. Although estimates of the prevalence of both sarcopenia and frailty differ depending on exact definitions used, recent estimates suggest the prevalence of sarcopenia and frailty in older adults in Europe range from 6.5 to 31% and 6.5–21%, respectively, increasing greatly with age with cohorts of 60–69 year olds at the lower end of the range and those over 80 years old at the higher end [8].

The role of nutrition in preventing or delaying the onset of sarcopenia and frailty is well acknowledged, with nutritional interventions and physical activity considered to be the most effective interventions to delay or reverse these conditions [9,10,11]. More specifically, high-quality protein intake combined with resistance exercise is considered the primary intervention method for the preservation of muscle mass and skeletal strength in maintaining functional autonomy and independence in older adults [5]. Considerations regarding optimum protein intake for older adults to reduce sarcopenia risk and anabolic resistance include not only total protein intake but also the practical aspects of providing dietary protein (i.e., source and quality of dietary proteins (as measured by digestible indispensable amino acid score (DIAAS)), timing and distribution of protein intake, and intake of protein-sparing energy [12,13].

A review of nutrient intakes and adequacy in older adults in Europe has shown that, while mean intakes of protein among older adults across Europe are above current recommended intakes (0.8 g/kg body weight per day), noteworthy proportions of older adults have inadequate total protein intakes [14,15]. Furthermore, it is well acknowledged that, with advancing age, significant physiological changes occur in the body that can impair the ability to absorb and utilise protein efficiently, which has led to ongoing debate on the appropriateness of current nutritional guidelines for older adults with respect to protein quantity and quality [13,16,17,18]. In parallel with an increased global emphasis to limit intake of high-biological-value animal protein (with more favourable DIAASs generally) and to consume more plant-based proteins instead, estimation of individual amino acid intake and sources in population groups is necessary in order to monitor and optimise the protein quality of the diet [19,20].

There are few nationally representative data available on amino acid intakes and sources for any population group, including that of older adults. To the best of the authors’ knowledge, the United States, Japan, and Korea are the only countries which have estimated amino acid intake in older adults using data from nationally representative dietary surveys [21,22,23], with no data available in European populations. For all three countries where data were available, essential amino acid intakes were above minimal population requirements. However, the authors of the Japanese study noted that almost 50% of the Japanese population were not meeting recommended levels of protein for the prevention of sarcopenia of 1.0–1.2 g/kg of body weight per day [13,24].

With regard to key dietary sources of individual amino acids, there are no nationally representative food consumption data available for older adults specifically. However, data are available for Korea (individual but for all age groups) and from studies of the food supply in Poland (from a household budget study) and Portugal (from a total diet study) [25,26]. Findings from Portugal and Poland were in agreement with each other, reporting red meat to be the most common source of amino acids in the diet, followed by white meat and fish in both countries [25,26]. However, for Korea, grains were found to be a key source of amino acids, together with meat and seafood [23].

The paucity of data on amino acid intakes and sources may be related to the limited availability of country level amino acid food composition data for inclusion in food consumption datasets and leaves a gap in fully understanding the protein quality in the diet of older adults. Hence, the aim of this study was to add to this very limited data on the intake of amino acids in the older adult diet by firstly updating the Irish Food Composition Database (IFCD) (2011) (www.iuna.net, accessed on 1 April 2021) with amino acid composition data, and secondly, to provide a dataset of nationally representative intakes and sources of amino acids in older adults living in Ireland, allowing us, for the first time, to estimate amino acid intake in an older adult population in Europe.

## 2. Materials and Methods

The data for this study are based on the Irish National Adult Nutrition Survey (NANS) (2008–2010) (www.iuna.net). The NANS was a nationally representative cross-sectional survey which collected data on the habitual food and beverage consumption of a stratified random sample of free-living adults aged 18–90 y (not pregnant/lactating) in the Republic of Ireland (n = 1500). The study was conducted according to the guidelines laid down in the Declaration of Helsinki and ethical approval was obtained from the Clinical Research Ethics Committee of the Cork Teaching Hospitals, University College Cork and the Human Ethics Research Committee of University College Dublin (ECM 3 (p) 04/11/08). Written informed consent was obtained from all participants and the final response rate for the survey was 60%. More details on the NANS can be found at www.iuna.net and the key methods relating to this study are outlined below.

### 2.1. Dietary Intake Assessment

Dietary intake data were collected at brand level using a 4-day semi-weighed food record which included at least one weekend day for all participants. Participants were asked to record detailed information regarding the amount, type, and brand of all food, beverages, and nutritional supplements consumed over the four-day period and, where applicable, the cooking methods used and details of any recipes of composite dishes. The researcher made three visits to the participant during the four-day period: a training visit to demonstrate how to complete the food record and how to use the weighing scales provided; a second visit 24–36 h into the recording period to review the food record, check for completeness, and clarify details regarding specific food descriptors and quantities; and a final visit one or two days after the recording period to check the last days and to collect the food record and scales. A hierarchal method was used to quantify the amount of each food/beverage consumed and which included direct weighing of the food by participants (46%), weights provided on product labels or from manufacturers (10%), use of a photographic food atlas (16%) [27], standard food portion sizes (11%) [28], household measures (11%), and estimated quantities (for a small number of foods based on participants’ previous recordings) (2%). Details of recipes of composite dishes were also recorded, and participants were asked to collect the packaging labels of all foods and beverages consumed over the recording period. Dietary intake data were analysed using WISP© (Weighed Intake Software Package) Version 3.0 (Tinuviel Software, Warrington, UK) based on data from the UK Composition of Foods database [29]. During the survey, modifications were made to the food composition database to include recipes of composite dishes, fortified foods, nutritional supplements, generic Irish foods that were commonly consumed, and new foods on the market.

### 2.2. Body Weight

Body weight was measured in duplicate (to the nearest 0.1 kg) for each participant in the NANS using a Tanita body composition analyser (BC-420MA (Tanita Ltd., Manchester, UK)), as part of a suite of anthropometric measurements taken by trained researchers in the participant’s own home.

### 2.3. Updating the IFCD (2011) with Amino Acid Values

The IFCD (2011) contains a dataset of nutritional information for 2552 unique food codes (foods, beverages, and nutritional supplements) from foods consumed by all adults (18–90 y) in the NANS. In the current study, these food codes (excluding nutritional supplements (n = 226)) were updated with amino acid content data. Initially, 22 amino acids (21 proteinogenic amino acids and 1 nonstandard amino acid, hydroxyproline) were considered for inclusion. Due to limitations in the standard analytical methodology used to treat asparagine, glutamine, and cysteine, these amino acids were excluded. During sample treatment, closely related amino acids can be partially destroyed and are therefore difficult to differentiate; thus, aspartic acid is calculated and reported as a sum of asparagine and aspartic acid, glutamic acid is reported as the sum of glutamine and glutamic acid [30], and cysteine is measured as its oxidised dimeric form cystine [31]. Hydroxyproline was omitted due to the lack of standardisation of available data between sources. Following these exclusions, the database was updated for the following amino acids for the nine essential amino acids (histidine, isoleucine, leucine, lysine, methionine, phenylalanine, threonine, tryptophan, and valine) and also for arginine, alanine, aspartic acid, glutamic acid, glycine, proline, and serine.

Updating of the dataset with amino acid values took place between May 2021 and February 2022 using the protocol outlined below, with a consensus on all decisions made by three researchers. A quantitative summary of sources used is presented in Table 1.

(1) If protein content per 100 g food was equal to zero, then each amino acid was given a value of zero.
(2) For foods where the protein content was greater than zero, in accordance with best practice from the WHO [32], other publicly available food composition databases were sourced, and amino acid values were matched by food descriptors.

Databases were consulted in the following order and as appropriate for the food group/type:

- US Department of Agriculture (USDA) FoodData Central [30];
- Canadian Nutrient File (CNF) [33];
- The Danish Food Composition Database (Frida V 4.2.2022) [34];
- Food Composition Database for Epidemiological Studies in Italy (BDA) [35];
- Standard Tables of Food Composition in Japan (MEXT) [36];
- FAO/INFOODS Global Food Composition Database for Fish and Shellfish (uFISH1.0) [37];
- Indian Food Composition Tables (IFCTs) [38].

In certain cases, where there was no published composition value for a food, the amino acid content of a similar product was used. For example, the amino acid values for ‘Nesquik cereal’ from the CNF database were assigned to ‘Aldi Harvest Morn’ cereal, as both products had similar ingredients and nutrient profiles.

(3) For recipes of composite dishes and products where an appropriate match was not available, but ingredient listings were available, the amino acid values were calculated from the ingredients (accounting for yield losses where appropriate). If a recipe contained a specific ingredient with no amino acid composition data available, the value of a similar ingredient was substituted to facilitate calculating the total amino acid content of the recipe. For example, all recipes containing the ingredient ‘Sauce, dry mix’ were substituted with AA values derived for ‘Stock cubes, chicken’ sourced from the MEXT database. In certain cases, where a recipe was the same as that of another, it was assigned the same amino acid composition values. Where entries differed in edible weight from those available in the databases, edible weights were compared using data taken from McCance and Widdowson’s ‘The Composition of Foods’, sixth and fifth editions, plus all nine supplemental volumes [29,39,40,41,42,43,44,45,46,47,48], which were used to generate the nutrient intake data for the IFCD. The amino acid composition of the food item was then calculated with new edible weights according to refuse values of the product. For example, food item. 14316, chicken, wing quarter, roasted, meat and skin, weighed with bone. Protein 13.1 g/100 g has the following refuse value:

- bone 48%
- total refuse 48%

Calculation = Original protein or AA value× refuse value = new protein or AA value

(4) For any foods for which there were no appropriate amino acid data available in other databases, and recipes could not be calculated from ingredient lists, analytical values were obtained from published papers [49,50].

Throughout each of the above steps, careful consideration was given to the calculation of edible and non-edible food portions, when assigning amino acid values to foods.

**Table 1:** Sources used for updating of the Irish Food Composition Database for amino acids.

Sources	Food Codes	
	n	%
Food items with a protein value of ‘0’	240	10.3
Food composition databases		
USDA Food Data Central	753	32.4
Canadian Nutrient File (CNF)	261	11.2
Frida V 4.2.2022	99	4.3
Food Composition Database for Epidemiological Studies in Italy (BDA)	49	2.1
Standard Tables of Food Composition in Japan (MEXT)	19	0.0
AO/INFOODS Global food composition database for fish and shellfish (uFISH1.0)	24	1.0
Indian Food Composition Tables (IFCTs)	4	0.2
Published papers		
Nigam & Singh (2014) (for Quorn products) [49]	2	0.1
Bonke et al. (2020) (for non-dairy alternative drink, oat) [50]	1	0.0
Calculated Data		
Recipes of composite dishes		
Recipes from the IFCD (2011) (with full ingredient listing)	720	31.0
New recipes developed to determine amino acid content of a food product	153	6.6
Total	2326	100

### 2.4. Mean Daily Intake of Amino Acids in Older Adults

For the purposes of the current study, data from a subgroup of older adults aged 65 years and over (n = 226) were used. Using SPSS© for Windows^™^ Version 26.0, the mean daily intake (MDI) of each amino acid was estimated for each individual by summing the total intake of each amino acid over the survey period and dividing by the number of recording days of the study (4 days). MDIs are presented as mean, SD, median, and 5th and 95th percentiles for absolute intakes (g/d), and mean and SD for intakes expressed per kg body weight (mg/kgbw) for the total population of older adults and by biological sex (male, female) and age group (65–74 y and 75–90 y). For amino acids where recommendations are available, recommended dietary allowance (RDA) values from the IOM are included in the tables as reference points, with the RDA being set as the value that will meet the needs of 97.5% of a healthy population group [51].

### 2.5. Key Sources of Amino Acids

The key food sources contributing to the intakes of each amino acid were estimated using the mean proportion method [52]. The mean proportion method provides information about the sources that are contributing to the nutrient intake ‘per person’ and is the preferred method when determining important food sources of a nutrient for individuals in a population group, as opposed to investigating the sources of a nutrient within the food supply.

### 2.6. Statistical Analysis

Differences in amino acid intakes between sexes (males, females) and age groups (65–74 y, 75–90 y) were assessed using independent sample *t*-tests, regardless of normality (due to the large sample size). As sample size increases, so does the robustness of *t*-tests to identify deviations from normality; thus, parametric tests are recommended for large samples [53]. To minimise type 1 errors (as a result of multiple testing), the Bonferroni adjustment was used by dividing the α level (0.05) by the number of comparisons (72 comparisons) [54]. Therefore, intakes were considered to be significantly different from each other if *p* < 0.001.

## 3. Results

Table 2 describes the MDI of amino acids (g/d, mg/kgbw/d) in adults aged 65 years and over in Ireland. The total mean daily amino acid intake for adults aged 65 years and older (sum of all individual amino acids) was found to be 76.2 g/d and 1.0 g/kgbw/d, respectively.

Regarding individual amino acids, the MDIs in g/d ranged from 0.9 g/d for tryptophan to 14.5 g/d for glutamic acid. When expressed per kg body weight, the MDIs of amino acids ranged from 12.1 mg/kgbw/d for tryptophan to 198 mg/kgbw/d for glutamic acid. For those amino acids where an established RDA was available (Histidine, Isoleucine, Leucine, Lysine, Threonine, Tryptophan, Tyrosine, Valine), the MDIs of amino acids in older adults in Ireland (adjusted for body weight) were above the respective RDAs proposed by the US IOM [55].

Table 3 and Table 4 describe the MDI of amino acids (g/d, mg/kgbw/d) in adults aged 65 years and over in Ireland, split by biological sex (Table 3) and by age group (Table 4). Total mean daily amino acid intake for adults aged 65 years and older was 1.03 g/kgbw/d for males, 0.97 g/kgbw/d for females, 1.01 g/kgbw/d for those aged 65–74 y, and 0.99 g/kgbw/d for those aged 75–90 y, with no significant differences observed in total amino acid intakes between sexes or age groups. Similarly, regarding intakes of individual amino acids, no significant differences were observed between sexes or age groups.

Table 5 describes the key sources of amino acids in adults aged 65 years and over in Ireland. The food group with the highest contribution to amino acid intake across all 18 amino acids was ‘meat and meat dishes’, contributing between 28 and 47% to individual amino acids. ‘Breads and rolls’, ‘milk and yoghurt’ (primarily milk), and ‘fish and fish dishes’ were also important contributors (up to 22% intake of individual amino acids each). While animal foods and products such as meat, fish, eggs, and dairy products were the predominant contributors to most amino acids, it is important to note that plant-based foods such as grains and cereal products provided a notable contribution (approx. 33%) to amino acids such as glutamic acid, cysteine, and proline.

## 4. Discussion

This study aimed to update the IFCD (2011) with data on amino acid composition and to estimate for the first time the intakes and dietary sources of amino acids in a nationally representative sample of older adults living in Ireland. The key outputs and findings of this study include a novel dataset of amino acid values (mg/100 g) for foods consumed by adults in Ireland based on the nationally representative NANS. Furthermore, this is the first study to estimate intakes and sources of amino acids for any nationally representative population group in Europe. For those amino acids where an established RDA was available, mean daily amino acid intakes (per kg body weight) in older adults in Ireland were shown to be above these values. The key dietary source for all amino acids examined was ‘meat and meat dishes’ (28–47%, range across amino acids) with ‘breads and rolls’, ‘milk and yoghurt’ (primarily milk), and ‘fish and fish dishes’ also being important contributors (up to 22% intake for individual amino acids).

### 4.1. Amino Acid Intakes in Older Adults—Comparison with Other Studies

Total mean daily amino acid intake for adults aged 65 years and older was found to be 1.0 g/kgbw/d (ranging from 0.99 to 1.03 g/kgbw/d across sex and age groups). Despite the growing interest in protein quality and amino acid composition of the diet, there are few available nationally representative estimates of amino acids in older adults with data only available from the US, Korea, and Japan. The findings of our study were remarkably similar to those of estimates in the US older adult population who reported total amino acid intakes for those aged 71–80 y of 1.07 g/kgbw/d for males and 1.01 gkgbw/d for females, and for those over 80 years, 1.04 g/kgbw/d for males and 1.01 g/kgbw/d for females [21]. Crude comparisons between our study and those of Japanese and Korean older adults showed that the mean daily intake of essential amino acids in older adults in Ireland (29.7 g/d) were comparable to that of older adults in Japan (35.3 g), but higher than that of older adults in Korea (18.5 g), which is reflective of the traditional rice-based diet in Korea, with a lower (yet increasing) contribution from animal products [22,23].

A key finding from our study, which was also in keeping with the findings from the US study, was that mean daily intakes of amino acids (per kg body weight) in the older adult population were above the RDAs proposed by the IOM [55]. It should, however, be noted that these RDAs are mainly derived from nitrogen balance studies and there is current discussion/debate on the updating of these DRVs, which were set two decades ago [13,56]. As proteins from different dietary sources have different digestibility rates, and food processing will alter digestion and free amino acid bioavailability, dietary recommendations should at least consider true ileal digestion of a particular protein (Digestible Indispensable Amino Acid Score) [20,57,58]. Furthermore, recommendations that optimise health outcomes for people of different ages and physiological states, including older adults, are also warranted [59,60].

### 4.2. Key Dietary Sources of Amino Acids in Older Adults—Comparison with Other Studies

In the current study, the key food group contributing to all amino acids examined was ‘meat and meat dishes’ (28–47%, range across individual amino acids) with ‘breads and rolls’, ‘milk and yoghurt’ (primarily milk), and ‘fish and fish dishes’ also being important contributors (up to 22% intake in individual amino acids). There is a lack of data available on dietary sources of individual amino acids specific to older adults. However, data are available for the total adult population in Korea, where key sources of amino acids in 2022 included grains and meats, with the intake of proline and alanine (mainly from grains) decreasing since 2010, and that of glycine and lysine (mainly from meat) increasing, suggesting a shift in protein quality, as meat and seafood are becoming more common in the Korean diet [23]. Other countries investigating the sources of amino acids in their food supply include Poland (household budget study) and Portugal (total diet study) [25,26]. Findings from both these studies were in keeping with our overall findings, reporting that meat and meat products, grain products, and milk and dairy products were important sources of amino acids within the diet, with meat and meat products contributing more than 40% of daily intake of glycine, lysine, alanine, histidine, arginine, threonine, methionine, aspartic acid, tryptophan, and isoleucine in the Polish diet, with similar findings from our study (>37%). With regard to plant sources of protein, our study showed that grains (including breads and cereal products) contributed approx. one third of the intake of cysteine, glutamic acid, and proline, which were slightly lower than that reported for Poland (34–41%). These findings highlight the current importance of animal products in terms of amino acid intake in the adult population but also acknowledge the contribution of plant proteins to the intake of certain amino acids; however, it is important to keep in mind the low digestibility of many plant proteins [12,20,57,58]. As dietary recommendations shift towards a more plant-based diet, in line with planetary health goals, plant-based proteins are gaining more prominence within the food industry, and it will be important to continue to monitor not only the amino acid intake and sources in the diet but also their true ileal digestion and bioavailability [12,20,57,58,61].

### 4.3. Public Health Implications

This study provides important data for the ongoing monitoring of protein and amino acid intakes in ageing populations. While current intakes of amino acids in older adults in Ireland appear to be adequate when compared to current recommendations (at least at mean population level), there is ongoing debate on the appropriateness of these guidelines for older adults, as they may not adequately account for the significant physiological changes that occur with advancing age [13,18,62]. With respect to protein and amino acids, these physiological changes include anabolic resistance, altered splanchnic metabolism and reduced postprandial availability of amino acids, diminished response of skeletal muscle to protein synthesis (MPS), and loss of proteostasis, which, individually and combined, can profoundly impact the muscle mass, functional capacity, and ultimately independence in an ageing population [41,46,47,48,49,50], with studies showing that a higher intake in essential amino acids is associated with greater muscle mass and strength in older adults [62,63,64].

While this study showed the role of plant-based protein sources such as cereals and breads as important contributors to amino acids such as cysteine, glutamic acid, and proline, the reliance on animal foods as predominant sources for most amino acids (including all the essential amino acids (histidine, isoleucine, leucine, lysine, methionine, phenylalanine, threonine, tryptophan and valine)) suggests that the sufficiency of many amino acids may be at risk if global advice to replace animal-based protein with plant-based substitutes translates into actual practice. Nutrition policies should therefore emphasise strategies to optimise protein quality through balanced inclusion of both animal and high-quality plant proteins. The updated IFCD (2011) provides an essential tool for informing evidence-based dietary guidelines and public health initiatives aimed at promoting healthy and sustainable ageing.

### 4.4. Strengths and Limitations

This is the first study to map amino acid values to a food composition dataset for nationally representative samples of older adults in Ireland (and Europe). A key strength of this study was the comprehensive mapping of the amino acid data to the IFCD (2011). The detailed mapping of amino acid values was further strengthened by the high-quality food consumption data, which were collected at brand level in addition to the collection of recipes of composite dishes at ingredient level. However, as with any database mapping study, it is important to acknowledge that potential error may have been introduced while mapping amino acid values from external databases and recipe calculations, and hence, these findings should be taken with appropriate caution. While the data in this study are based on food consumption data collected between 2008 and 2010 and dietary patterns may have changed within this time, the detailed mapping protocol devised as part of the current study will facilitate the updating of any new foods that may arise with new food consumption patterns and also foods that are consumed by other population groups. A limitation in the dietary analysis is the use of mean daily intakes (average intake over 4 days) as opposed to usual intakes, which account for the variability in daily intake and provide a more stable and reliable measure of nutrient consumption over time, particularly at the upper or lower ends of distribution.

## 5. Conclusions

This study represents the first nationally representative assessment of amino acid intakes and dietary sources among older adults in Europe. Findings indicate that, at mean population level, total and individual amino acid intakes in older adults in Ireland are above current recommendations, suggesting that protein and amino acid quantities are sufficient within this population. However, further analysis using usual intake modelling would be helpful to determine if there is a proportion of the population with inadequate intakes of any individual amino acid. Furthermore, the dominant contribution of animal-based foods, particularly ‘meat and meat dishes’, highlights a nutritional dependency that may be challenged by evolving dietary recommendations favouring plant-based diets. The updated Irish Food Composition Database, now inclusive of amino acid data, will enable future dietary surveillance and modelling (including scenario analyses for plant-forward shifts).

## Figures and Tables

**Table 2:** Mean daily intake of amino acids in a representative sample of older adults in Ireland aged 65 years and over.

	Total Population (n = 226)										
	g/d					mg/kgbw/d					
	Mean	SD	Median	5th Percentile	95th Percentile	RDA *	Mean	SD	Median	5th Percentile	95th Percentile
Histidine	2.1	0.7	2.0	1.2	3.3	14	28.7	27.0	9.7	15.7	44.3
Isoleucine	3.4	1.0	3.2	1.9	5.1	19	46.3	43.1	15.3	26.8	73.1
Leucine	5.8	1.8	5.6	3.2	9.0	42	79.7	74.7	26.5	44.9	125
Lysine	5.5	4.0	4.9	3.0	8.4	38	75.0	68.3	53.4	40.2	118
Methionine	1.8	0.6	1.7	1.0	2.7	-	24.2	22.4	8.2	13.3	38.6
Phenylalanine	3.3	1.0	3.1	0.9	5.1	-	45.2	42.4	14.7	25.7	71.1
Threonine	3.0	0.9	2.9	1.7	4.6	20	41.1	38.8	13.5	23.4	65.6
Tryptophan	0.9	0.3	0.8	0.5	1.3	5	12.1	11.3	3.9	6.8	18.8
Valine	3.9	1.2	3.7	2.2	5.9	24	53.4	49.7	17.6	31.1	83.5
Alanine	3.6	1.1	3.5	2.0	5.6	-	48.6	45.9	16.0	27.0	75.9
Arginine	4.1	1.2	3.9	2.3	6.4	-	55.9	53.0	18.6	29.5	88.5
Aspartic acid	6.5	2.0	6.4	3.7	10.1	-	89.3	86.0	28.9	51.1	139
Cystine	1.0	0.3	1.0	0.6	1.6	-	13.6	12.8	4.5	7.7	21.1
Glutamic acid	14.5	4.3	13.8	8.2	21.9	-	198	184	64.8	115	312
Glycine	3.1	1.0	3.1	1.7	4.9	-	42.9	40.5	14.2	22.5	69.1
Proline	4.1	1.2	3.9	2.3	6.4	-	67.8	61.6	30.8	37.1	111
Serine	3.4	1.0	3.2	1.9	5.3	-	46.9	44.3	15.4	27.1	74.5
Tyrosine	2.6	0.8	2.5	1.5	4.0	5	35.4	33.4	11.9	20.0	56.2

Abbreviations: g = gram, SD = Standard Deviation, mg = milligram, kg = kilogram, bw = bodyweight. * Recommended dietary allowance (IOM, 2005). - No RDA available.

**Table 3:** Mean daily intake of amino acids in a nationally representative sample of older adults in Ireland aged 65 years and over, split by sex.

	Males *								Females							
	g/d					mg/kgbw/d			g/d					mg/kgbw/d		
	Mean	SD	Median	5th	95th	RDA *	Mean	SD	Mean	SD	Median	5th	95th	RDA	Mean	SD
Histidine	2.4	0.7	2.3	1.3	3.6	14	29.4	28.6	1.9	0.5	1.8	1.1	2.8	14	28.1	26.2
Isoleucine	3.8	1.1	3.7	2.0	5.8	19	46.8	44.1	3.1	0.8	3.0	1.8	4.7	19	46.0	42.1
Leucine	6.5	1.9	6.2	3.5	9.8	42	80.9	77.8	5.3	1.4	5.1	3.1	7.9	42	78.7	71.6
Lysine	6.3	5.6	5.7	3.2	9.3	38	79.9	71.3	4.8	1.3	4.7	2.8	7.2	38	70.9	66.9
Methionine	2.0	0.6	1.9	1.1	3.0	-	24.6	23.4	1.6	0.4	1.5	1.0	2.5	-	23.8	21.9
Phenylalanine	3.7	1.1	3.5	2.0	5.5	-	45.7	43.5	3.0	0.8	3.0	1.8	4.4	-	44.7	41.0
Threonine	3.3	1.0	3.3	1.8	5.1	20	41.5	40.0	2.7	0.8	2.7	1.6	4.1	20	40.8	37.9
Tryptophan	1.0	0.3	1.0	0.5	1.5	5	12.2	11.6	0.8	0.2	0.8	0.5	1.1	5	11.9	10.9
Valine	4.3	1.3	4.2	2.3	6.4	24	53.8	51.7	3.5	0.9	3.5	2.1	5.3	24	52.9	48.2
Alanine	4.0	1.2	3.9	2.2	6.1	-	49.7	47.9	3.2	0.9	3.2	1.8	4.7	-	47.8	45.2
Arginine	5.5	2.2	5.3	2.7	8.2	-	68.4	63.4	3.7	1.1	3.7	2.2	5.5	-	55.5	52.2
Aspartic acid	7.2	2.2	7.2	4.1	10.7	-	90.0	88.8	5.9	1.6	5.9	3.4	8.8	-	88.7	83.7
Cystine	1.1	0.3	1.1	0.7	1.8	-	13.9	13.4	0.9	0.2	0.9	0.5	1.4	-	13.4	12.1
Glutamic acid	16.1	4.6	16.1	8.9	24.2	-	202	193	13.1	3.5	12.4	8.0	19.8	-	196	178
Glycine	3.5	1.0	3.4	1.8	5.4	-	43.8	41.6	2.8	0.8	2.8	1.7	4.3	-	42.1	39.0
Proline	5.5	2.2	5.3	2.7	8.2	-	68.4	63.4	4.5	2.0	4.2	2.6	6.6	-	67.3	59.4
Serine	3.8	1.1	3.7	2.0	5.7	-	47.5	46.0	3.1	0.8	3.1	1.8	4.7	-	46.4	41.5
Tyrosine	2.9	0.9	2.8	1.5	4.6	5	36.0	34.6	2.3	0.7	2.3	1.5	3.6	5	35.0	32.0

Abbreviations: g = gram, SD = Standard Deviation, mg = milligram, kg = kilogram, bw = bodyweight. No statistical differences (*p* < 0.001) were found between males and females for any amino acid intake values via independent samples *t*-tests and adjusted for multiple testing. * Recommended dietary allowance (IOM, 2005). - No RDA available.

**Table 4:** Mean daily intake of individual amino acids in a nationally representative sample of older adults in Ireland aged 65 years and older, split by age group (65–74 y and 75–90 y).

	65–74 y (n = 149)								75–90 y (n = 77)							
	g/d					mg/kgbw/d			g/d					mg/kgbw/d		
	Mean	SD	Median	5th	95th	RDA *	Mean	SD	Mean	SD	Median	5th	95th	RDA	Mean	SD
Histidine	2.0	2.1	0.7	1.3	3.4	14	29.1	27.5	2.0	1.8	0.6	1.1	3.1	14	27.9	25.1
Isoleucine	3.5	3.3	1.0	2.1	5.4	19	46.8	43.3	3.2	2.9	1.0	1.7	4.8	19	45.3	42.1
Leucine	6.0	5.6	1.8	3.6	9.3	42	80.5	75.0	5.5	5.0	1.7	3.0	8.4	42	78.1	73.1
Lysine	5.8	5.1	4.8	3.1	8.9	38	77.4	68.0	4.9	4.7	1.5	2.8	7.4	38	70.0	68.6
Methionine	1.8	1.7	0.6	1.1	2.9	-	24.4	22.4	1.7	1.6	0.5	1.0	2.6	-	23.7	22.2
Phenylalanine	3.4	3.2	1.0	2.0	5.3	-	45.7	42.7	3.1	2.8	0.9	1.7	4.8	-	44.2	41.2
Threonine	3.1	3.0	0.9	1.8	4.9	20	41.6	39.3	2.8	2.6	0.9	1.6	4.4	20	40.1	37.2
Tryptophan	0.9	0.9	0.3	0.5	1.4	5	12.2	11.3	0.8	0.8	0.3	0.5	1.3	5	11.8	11.2
Valine	4.0	3.8	1.2	2.4	6.2	24	53.9	50.5	3.7	3.4	1.1	1.9	5.7	24	52.3	49.2
Alanine	3.7	3.5	1.1	2.1	5.7	-	49.1	46.4	3.3	3.2	1.0	1.8	5.2	-	47.7	44.1
Arginine	4.2	4.1	1.2	2.5	6.6	-	56.2	53.9	3.8	3.7	1.2	2.1	6.1	-	55.2	49.6
Aspartic acid	6.8	6.6	2.0	4.0	10.3	-	90.7	86.6	6.0	5.9	1.8	3.3	9.7	-	86.4	83.1
Cystine	1.0	1.0	0.3	0.6	1.7	-	13.8	13.0	0.9	0.9	0.3	0.5	1.4	-	13.3	12.4
Glutamic acid	14.9	14.4	4.3	8.9	22.1	-	199	190	13.6	13.1	4.2	7.2	21.3	-	196	180
Glycine	3.2	3.1	1.0	1.8	5.3	-	43.2	40.9	2.9	2.9	0.9	1.7	4.6	-	42.4	39.0
Proline	5.0	4.7	1.5	2.9	7.6	-	66.4	61.7	4.9	4.4	2.9	2.4	7.6	-	70.7	61.5
Serine	3.6	3.3	1.0	2.1	5.4	-	47.4	44.7	3.2	3.0	1.0	1.6	5.0	-	45.8	43.1
Tyrosine	2.7	2.5	0.8	1.6	4.2	5	35.9	33.6	2.4	2.3	0.7	1.3	3.7	5	34.4	32.9

Abbreviations: g = gram, SD = Standard Deviation, mg = milligram, kg = kilogram, bw = bodyweight. No statistical differences (*p* < 0.001) were found between males and females for any amino acid intake values via independent samples *t*-tests and adjusted for multiple testing. Amino acids listed in order from largest to smallest overall intakes. * Recommended dietary allowance (IOM, 2005). - No RDA available.

**Table 5:** Contribution of food groups (%) to mean daily intakes of individual amino acids in a nationally representative sample of older adults in Ireland aged 65 years and older.

	Hist	Ile	Leu	Lys	Met	Phe	Thr	Trp	Val	Ala	Arg	Asp	Cys	Glu	Gly	Pro	Ser	Tyr
	%																	
Meat and meat dishes	44.2	39.2	38.5	45.9	43.1	34.8	40.6	35.7	36.5	45.7	44.1	37.0	33.8	31.4	46.8	27.8	33.0	37.5
*Red meat (including dishes)*	*20.2*	*17.4*	*17.8*	*21.3*	*19.8*	*15.8*	*18.7*	*15.6*	*16.7*	*20.5*	*20.3*	*15.5*	*14.2*	*14.4*	*20.1*	*11.9*	*15.0*	*17.4*
*Poultry (including dishes)*	*11.1*	*11.2*	*10.0*	*12.2*	*11.7*	*9.2*	*10.7*	*10.0*	*9.6*	*12.0*	*11.5*	*10.5*	*9.7*	*8.2*	*12.7*	*7.3*	*8.2*	*10.0*
*Processed meat*	*12.8*	*10.6*	*10.7*	*12.4*	*11.7*	*9.8*	*11.2*	*10.1*	*10.2*	*13.2*	*12.3*	*11.0*	*9.9*	*8.7*	*14.0*	*8.5*	*9.7*	*10.2*
Bread and rolls	11.0	11.6	12.3	6.0	10.0	15.0	10.4	14.0	12.0	10.5	10.7	8.3	19.9	21.3	12.2	22.1	14.5	11.2
*White bread and rolls*	*3.9*	*4.3*	*4.5*	*1.9*	*3.5*	*5.5*	*3.7*	*4.9*	*4.4*	*3.6*	*3.7*	*3.0*	*7.6*	*8.2*	*4.5*	*8.4*	*5.4*	*3.7*
*Wholemeal, brown bread, and rolls*	*6.1*	*6.2*	*6.6*	*3.3*	*5.4*	*8.0*	*5.7*	*7.8*	*6.4*	*5.9*	*5.9*	*4.5*	*10.5*	*11.1*	*6.6*	*11.5*	*7.6*	*6.2*
*Other breads*	*1.1*	*1.1*	*1.3*	*0.7*	*1.1*	*1.5*	*1.0*	*1.4*	*1.2*	*1.0*	*1.2*	*0.8*	*1.8*	*2.0*	*1.1*	*2.2*	*1.5*	*1.3*
Milk and yoghurt	10.9	12.6	13.1	13.3	11.8	11.8	11.9	11.4	13.2	7.8	6.9	10.2	7.1	11.7	5.6	16.3	13.0	14.1
*Milk*	*9.2*	*10.4*	*10.9*	*11.0*	*9.8*	*9.8*	*9.6*	*9.8*	*16.3*	*6.4*	*5.8*	*8.5*	*6.1*	*9.9*	*4.5*	*13.2*	*10.8*	*11.7*
*Yoghurt*	*1.7*	*2.2*	*2.2*	*2.3*	*2.0*	*2.0*	*2.2*	*1.7*	*3.6*	*1.4*	*1.1*	*1.7*	*1.0*	*1.8*	*1.1*	*3.1*	*2.2*	*2.4*
Fish and fish dishes	9.3	9.8	9.7	11.9	12.1	8.6	10.1	9.0	9.6	11.7	11.7	10.4	7.5	7.3	11.0	6.0	8.7	9.4
Breakfast cereals	4.1	4.9	5.3	3.6	3.9	6.0	5.2	5.5	5.2	4.4	4.8	8.3	8.1	5.8	5.9	5.5	5.9	5.1
Vegetables and vegetable dishes	2.7	2.8	2.6	2.7	1.9	3.0	3.7	2.8	2.9	3.1	3.8	4.1	3.4	3.4	2.7	2.0	3.0	2.3
Cheeses	3.4	3.8	3.8	3.7	3.8	3.8	2.9	3.8	4.1	2.2	2.0	2.6	1.6	3.3	1.6	5.2	3.9	4.9
Potato and potato products	2.7	3.2	2.8	3.0	2.4	3.6	3.3	4.6	4.0	2.5	3.1	9.5	3.2	3.1	2.6	2.1	3.3	3.6
Biscuits, cakes, and pastries	1.6	1.8	1.9	1.1	1.6	2.2	1.6	2.1	1.9	1.7	1.8	1.6	2.8	2.4	1.8	2.8	2.3	1.7
Eggs and egg dishes	2.6	3.6	3.3	3.1	3.9	3.6	3.5	3.4	3.7	3.6	3.4	3.6	4.9	2.1	2.5	2.0	4.8	3.5
Grains, rice, pasta, and savouries	1.5	1.5	1.7	1.1	1.4	1.9	1.5	1.9	1.7	1.6	1.7	1.4	2.3	2.1	1.6	1.9	1.8	1.6
Soups, sauces, and misc. foods	1.1	1.0	1.0	1.0	0.8	1.0	1.3	1.0	1.1	1.2	1.2	1.3	1.1	2.0	1.4	1.1	1.1	0.9
Ice creams and chilled desserts	1.5	1.8	1.8	1.7	1.6	1.8	1.6	1.9	1.8	1.4	1.4	1.5	1.6	1.7	1.3	2.2	1.9	1.9
Fruit and fruit dishes	1.7	0.8	0.9	0.9	0.7	1.1	0.9	1.0	1.1	1.3	1.6	2.6	1.0	0.9	1.5	1.2	1.2	0.6
Nuts, seeds, herbs, and spices	0.5	0.5	0.5	0.3	0.4	0.6	0.5	0.7	0.5	0.6	1.1	0.6	0.6	0.6	0.8	0.4	0.6	0.5
Other	1.2	0.9	0.9	0.8	0.7	1.1	1.0	1.3	0.7	0.8	0.5	1.0	1.6	1.0	1.0	1.5	1.1	1.2

Abbreviations: Hist = Histidine, Ile = Isoleucine, Leu = Leucine, Lys = Lysine, Met = Methionine, Phe = Phenylalanine, Thr = Threonine, Trp = Tryptophan, Val = Valine, Ala = Alanine, Arg = Arginine, Asp = Aspartic Acid, Cys = Cysteine, Glu = Glutamic Acid, Pro = Proline, Ser = Serine, Tyr = Tyrosine. Italics: denotes subgroups of larger food groups.

## Data Availability

Restrictions apply to the availability of these data. Data were obtained from the Irish Universities Nutrition Alliance and are available [from the authors/at www.iuna.net] with permission from the IUNA data access committee.
